# Blue Light Induces RPE Cell Necroptosis, Which Can Be Inhibited by Minocycline

**DOI:** 10.3389/fmed.2022.831463

**Published:** 2022-04-26

**Authors:** Weilin Song, Ruilin Zhu, Wenna Gao, Chen Xing, Liu Yang

**Affiliations:** Department of Ophthalmology, Peking University First Hospital, Beijing, China

**Keywords:** necroptosis, cell death, retinal pigment epithelium, minocycline, blue light, retinal degeneration

## Abstract

**Purpose:**

Damage to and death of the retinal pigment epithelium (RPE) are closely related to retinal degeneration. Blue light is a high-energy light that causes RPE damage and triggers inflammatory responses. This study investigates whether blue light induces RPE necroptosis, explores pharmacologic therapy and specific mechanisms, and provides hints for research on retinal degeneration.

**Methods:**

The human RPE cell line ARPE-19 was cultured and subjected to blue light insult *in vitro*. Annexin V/PI was used to evaluate RPE survival. Minocycline was applied to inhibit the death of RPE. Proteomic measurement was used to analyze protein expression. Inhibitors of necroptosis and apoptosis were applied to assess the death mode. Immunofluorescence of protein markers was detected to analyze the mechanism of cell death. Subcellular structural changes were detected by transmission electron microscopy. Reactive oxygen species (ROS) was tested by DCFH-DA. Mitochondrial membrane potential (Δψ_m_) was detected by JC-1. BALB/c mice received bule light exposure, and RPE flatmounts were stained for verification *in vivo*.

**Results:**

Blue light illumination induced RPE death, and minocycline significantly diminished RPE death. Proteomic measurement showed that minocycline effectively mitigated protein hydrolysis and protein synthesis disorders. Necroptosis inhibitors (Nec-1s, GSK-872) increased the survival of RPE cells, but apoptosis inhibitors (Z-VAD-FMK) did not. After blue light illumination, high-mobility group box-1 (HMGB1) was released from the nucleus, receptor-interacting protein kinase 3 (RIPK3) aggregated, and mixed-lineage kinase domain-like protein (MLKL) increased in the RPE. The application of minocycline alleviated the above phenomena. After blue light illumination, RPE cells exhibited necrotic characteristics accompanied by destruction of cell membranes and vacuole formation, but nuclear membranes remained intact. Minocycline improved the morphology of RPE. Blue light increased ROS and decreased Δψ_m_ of RPE, minocycline did not reduce ROS but kept Δψ_m_ stable. *In vivo*, HMGB1 release and RIPK3 aggregation appeared in the RPE of BALB/c mice after blue light illumination, and minocycline alleviated this effect.

**Conclusions:**

Blue light exposure causes RPE necroptosis. Minocycline reduces the death of RPE by keeping Δψ_m_ stable, inhibiting necroptosis, and preventing HMGB1 release. These results provide new ideas for the pathogenesis and treatment of retinal degeneration.

## Introduction

RPE is a special epithelial cell located between the neuroretina and choroid that plays a crucial role in maintaining normal visual function. RPE cells can absorb light, protect against photooxidation, exchange heat, phagocytize the outer segment of the photoreceptor, participate in vitamin-A metabolism, secrete vascular endothelial growth factors, participate in the formation of the blood–retina outer barrier, and provide oxygen and nutrients from the choroid to the outer retina (1–3). Owing to the complex physiological functions of RPE cells, damage to and death of RPE cells are closely related to retinal degeneration such as retinitis pigmentosa (RP), Best disease, age-related macular degeneration (AMD), and Stargardt disease.

The structures of the eyes, including the cornea, aqueous humor, lens, and vitreous humor, absorb photons of different wavelengths sequentially. The visible light component (380–780 nm) of optical radiation can reach the retina, and the blue light component (400–500 nm) is particularly important because of its high energy (4, 5). The blue light part of 400 to 460 nm is considered to cause potential phototoxic retinal damage (4, 6). A wavelength of approximately 440 nm has been shown to be an excitation peak that damages photoreceptors and RPE function (6). In rhesus, 441 nm blue light-induced photochemical lesions originate in the RPE (7). Light-emitting diodes (LEDs) are widely used in daily life. Compared with traditional light sources, LED light sources emit more blue light, peaking at 435 to 460 nm (4, 8). This condition can lead to a range of health problems including retinal photochemical damage and disorder of circadian rhythms (4, 9, 10). In this study, we used blue light to induce RPE cell damage, studied the mode of RPE death, and explored therapeutic strategies and specific mechanisms.

Cell death is now divided into two categories: accidental cell death (ACD) and regulated cell death (RCD) (11). ACD is caused by severe lesions such as a consequence of burns and is immediate and insensitive to pharmacologic treatment (11). RCD means that cell death can be genetically regulated and modified by pharmacologic or genetic intervention (11–13). Apoptosis is a type of traditional RCD. Necrosis used to be considered passive cell death that could not be regulated. Currently, some types of cell death possess necrotic manifestations, such as necroptosis, which is regulated by RIPK1/3 and is a member of RCD. In the process of necroptosis, RIPK1 recruits RIPK3 to form the necrosome. MLKL is then recruited and activated by RIPK3 and ultimately executes necroptosis (14). Research of Murakami et al. (15) showed that in dsRNA-induced retinal degeneration, necroptosis of RPE cells was crucial and participated in damage-associated molecular patterns (DAMPs)-mediated inflammation. The dysfunction and death mode of RPE in retinal degenerations needs further study.

As a second-generation semisynthetic tetracycline, minocycline has antibiotic properties and can cross the blood-brain barrier. In addition to its antibiotic properties, minocycline has been found to exhibit neuroprotective and anti-inflammatory properties (16–18). Minocycline has been proven to be neuroprotective in various disease models. These disease models contain hemorrhagic and ischemic stroke, spinal cord injury, neuropathic pain, multiple sclerosis (MS), Alzheimer's disease (AD), Huntington's disease, Parkinson's disease, and amyotrophic lateral sclerosis (ALS) (17, 19).

In this study, we adopted blue light exposure to induce human RPE cell damage and death, assessed the death mode of RPE cells induced by the blue light, and sought corresponding pharmacologic treatment. We applied minocycline to inhibit necroptosis of RPE cells and explored the mechanism, demonstrating that minocycline had an excellent rescue effect on dying RPE cells.

## Materials and Methods

### Reagents and Antibodies

Minocycline hydrochloride, Nec-1s, GSK-872, and Z-VAD-FMK were purchased from MedChemExpress (Monmouth Junction, NJ, USA). Anti-HMGB1 antibody and anti-rabbit IgG (H+L), F(ab')2 fragment (Alexa Fluor 488 Conjugate) were purchased from Cell Signaling Technology (Danvers, MA, USA). Anti-ZO-1, anti-RIPK3 and anti-MLKL antibodies were purchased from Proteintech (Rosemont, IL, USA). Mitochondrial membrane potential assay kit with JC-1 and ROS assay kit were purchased from Beyotime Biotechnology (Shanghai, China). DMEM/F12 and DMSO were purchased from Sigma-Aldrich (St. Louis, MO, USA). Fetal bovine serum was purchased from Gibco (Logan, UT, USA). Dead cell apoptosis kit with Annexin V Alexa Fluor 488 & Propidium Iodide (PI), goat anti-rabbit IgG (H+L) (Alexa Fluor 488), and goat anti-mouse IgG (H+L) (Alexa Fluor 568) were purchased from Invitrogen (Carlsbad, CA, USA).

### Cell Culture and Blue Light Illumination

ARPE-19 cells were cultured in DMEM/F12 supplemented with 10% FBS and 1% penicillin-streptomycin at 37°C in 5% CO_2_. After the medium was replaced, the corresponding drugs (minocycline, Nec-1s, GSK-872, and Z-VAD-FMK) were added to the medium and preincubated for 30 min at 37°C in 5% CO_2_. Then, the cells were exposed to 2200 lux blue light (440–445 nm wavelength) at 37°C in 5% CO_2_. The time of blue light exposure was determined according to the experimental needs.

### Annexin V/PI Staining

ARPE-19 cells were harvested and washed in cold PBS. Then, the cells were resuspended in 1X annexin-binding buffer. The cell density was 1 × 10^6^ cells/ml. Afterward, 5 μl Alexa Fluor 488 annexin V and 1 μl 100 μg/ml PI working solution were added to each 100 μl of cell suspension. The cells were incubated at room temperature for 15 min. Then, 400 μl of 1X annexin-binding buffer was added to the cell suspension. Next, stained cells were analyzed by flow cytometry (Calibur, BD, USA).

### Mass Spectrometry-Based Proteomic Measurement

For each group of cells, three biological replicates were prepared. Proteins were extracted from each group of cells. One hundred micrograms of protein sample were mixed with 100 μl of 8 M urea 0.1 MTtris/HCl solution, centrifuged for 15 min at 14,000 g, and repeated twice. Then, 100 μl 8 M urea 0.1 MTtris/HCL solution was added, and 10 μl 0.05 M TCEP solution was added, incubated at 37°C for 1 h. Then, 10 μl of 0.1 M IAA was added and incubated at 37°C for 1 h. The sample was centrifuged for 15 min at 14,000 g. The sample was washed twice with 50 mM ammonium bicarbonate. Trypsin (1 μg) was dissolved in 100 μl of 50 mM ammonium bicarbonate, and then the mixed liquid was added. The sample was incubated at 37°C overnight, and the peptide fragments were collected by centrifugation. LC–MS/MS analysis was performed with a mass spectrometer (Q-Exactive HF, Thermo Scientific, USA). MaxQuantsoftware (version 1.4.1.2) was used for data analysis.

### Transmission Electron Microscopy

ARPE-19 cells were trypsinized and washed by PBS. Then, the cells were fixed in 2.5% glutaraldehyde. The cells were postfixed in OsO_**4**_, dehydrated in ethanol, and embedded in epoxy resin. Ultrathin sections were made and then stained with uranium acetate and lead citrate double staining. These specimens were observed with transmission electron microscopy (JEM1400PLUS, JEOL, Japan).

### ROS Measurement

The culture medium was removed, and 10 μM DCFH-DA (diluted in serum-free culture medium) was added. ARPE-19 cells were incubated at 37°C in 5% CO_2_ for 25 min. Then, the cells were washed with serum-free cell culture medium three times to fully remove the non-intracellular DCFH-DA. The cells were collected, and the fluorescence intensity was detected by flow cytometry (Calibur, BD, USA).

### Mitochondrial Membrane Potential Detection

The supernatant was removed, and JC-1 dye solution (1×) was added and incubated at 37°C in 5% CO_2_ for 20 min. Then, the cells were washed twice with JC-1 buffer solution. The cell culture medium was added again, and fluorescence images were taken via laser confocal microscopy (TCS-SP8 STED 3X, Leica, Germany).

### Immunofluorescence

ARPE-19 cells of different groups were fixed with 4% paraformaldehyde for 20 min and blocked with 0.3% Triton X-100 and 5% goat serum for 30 min. Primary antibody was added and incubated at 4°C overnight. After washing with PBS, the cells were incubated with secondary antibodies for 1 h at room temperature. Next, the cells were stained with DAPI and mounted in antifade mounting medium. Fluorescence images were acquired via laser confocal microscopy (TCS-SP8 STED 3X, Leica, Germany).

### Animals and Blue Light Illumination

BALB/c mice (8 to 10 weeks, male) were used in this study. Mice received 24 h dark adaptation before blue light exposure. The minocycline group received intraperitoneal injection of minocycline 50 mg/kg 1 h before blue light exposure, and 12 h after blue light exposure. The blue light group received intraperitoneal injection of PBS as a control. Their pupils were dilated with eye drops containing 0.5% tropicamide and 0.5% phenylephrine (Santen Pharmaceutical, Osaka, Japan) before blue light exposure. The mice were exposed to 4500 lux blue light (440–445 nm wavelength) for 1 h.

### RPE Flatmount Staining

The eyes of the mice were enucleated 24 h after blue light illumination. The anterior segment and the neuroretina were removed and fixed in 4% PFA for 1 h. After washing with PBS, the eyecup was blocked with 0.3% Triton X-100 and 5% goat serum for 30 min, and primary antibody was added and incubated at 4°C overnight. The flatmounts were incubated with secondary antibodies for 1 h at room temperature after washing with PBS. Next, the flatmounts were stained with DAPI and mounted in antifade mounting medium. Fluorescence images were acquired via laser confocal microscopy (TCS-SP8 STED 3X, Leica, Germany).

## Results

### Blue Light Induces Death of RPE Cells, Which Can Be Inhibited by Minocycline

**Figure 1:**
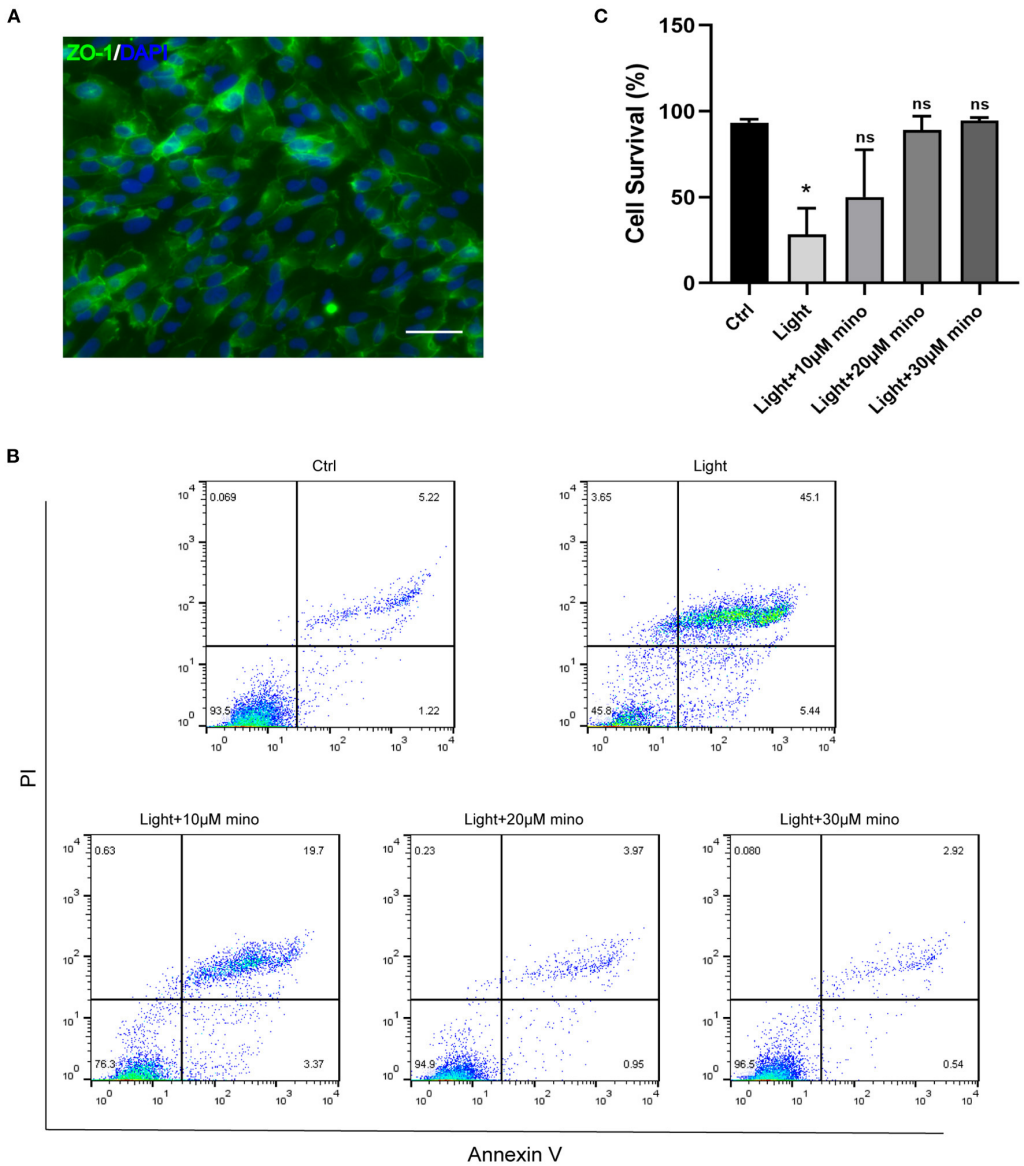
Blue light causes death of RPE cells, and minocycline protects RPE cells from blue light-induced death. **(A)** ZO-1 immunofluorescence staining of ARPE-19 cells indicated formation of tight junctions. Scale bar: 50 μm. **(B)** Detection of ARPE-19 cell viability with Annexin V/PI. With increasing minocycline doses, death of ARPE-19 cells induced by blue light decreased significantly. ARPE-19 cells received blue light exposure for 2 h and were further cultured for 2 h before Annexin V/PI detection. **(C)** Quantitative analysis of **(B)** (*n* = 3). *p < 0.05.

In this study, we used blue light at 440–445 nm to damage ARPE-19 cells. As a cell line derived from human RPE, ARPE-19 has excellent RPE characteristics. ZO-1 immunofluorescence staining of ARPE-19 cells was positive, which indicated the formation of tight junctions among the cells (Figure 1A). In addition to antibiotic properties, minocycline has been confirmed to have neuroprotective effects in various injury and neurodegenerative models. Minocycline has not been reported to be used to protect RPE cells from light damage. After the addition of minocycline, the ARPE-19 cellular state improved significantly. Flow cytometry confirmed that a large number of cells died after exposure to blue light. Most of the dead cells were located in the upper right quadrant of the flowchart, indicating that the cells were Annexin V/PI double-staining positive (Figure 1B). After application of minocycline, the number of Annexin V/PI double-stained positive cells decreased dramatically, and the cell survival percentage increased. As the dose of minocycline increased, the cell survival percentage rose successively (Figure 1B). The application of 20 or 30 μM minocycline substantially increased the percentage of surviving cells, indicating the protective effect of minocycline on blue-light-damaged RPE cells (Figure 1C).

**Figure 2:**
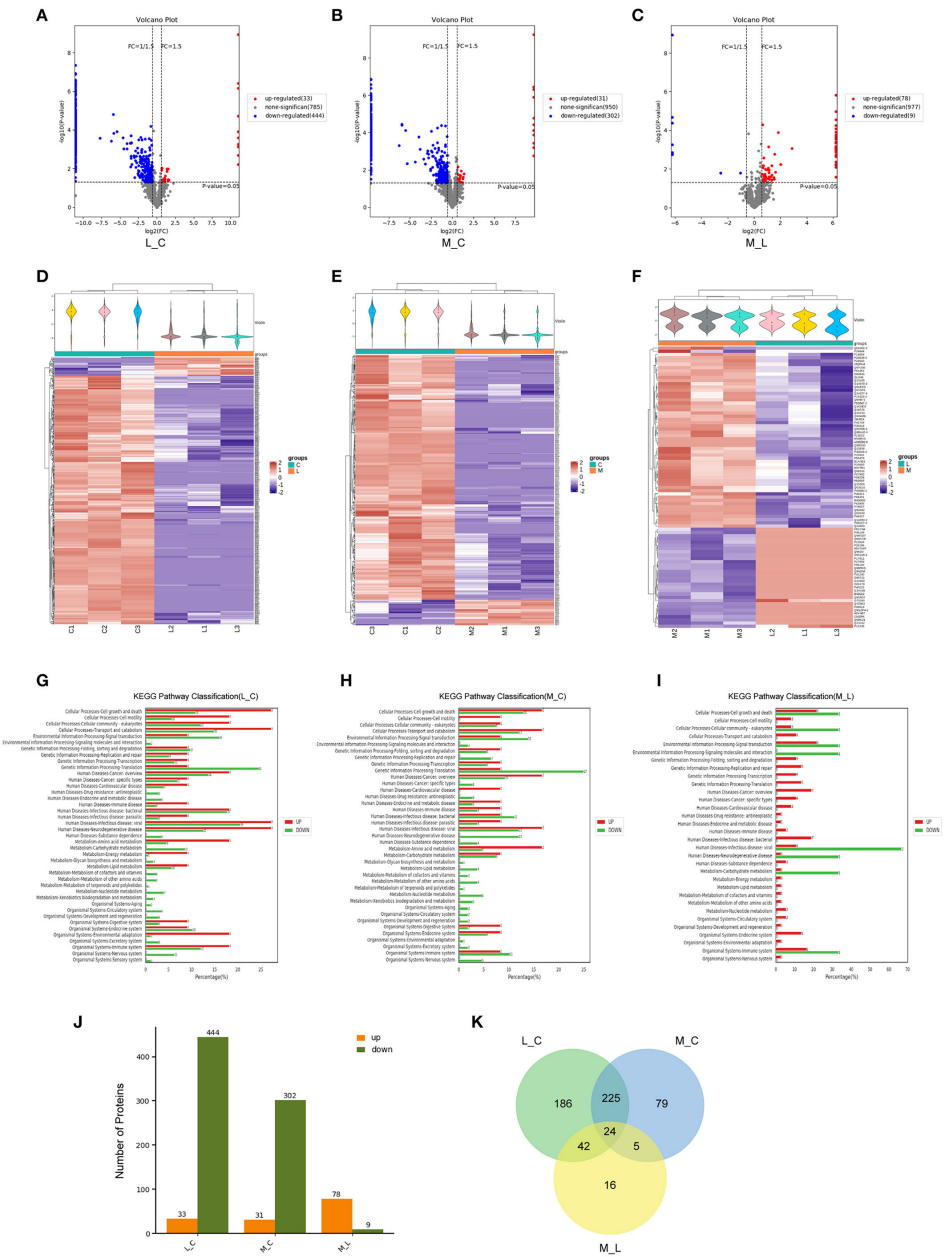
Mass spectrometry-based proteomic analysis of cell damage caused by blue light and rescue effect of minocycline *in vitro*. ARPE-19 cells received blue light exposure for 2 h and were further cultured for 2 h before follow-up experiments. **(A–C)** Volcano plots of different groups. Light damage led to decrease in expression of large number of proteins, which was alleviated by minocycline. **(D–F)** Violin plot and clustering heatmap. Cluster analysis of protein expression in different groups. **(G–I)** Distribution map of upregulated and downregulated proteins at KEGG Level 2 in three groups. **(J)** Number of upregulated and downregulated proteins in different groups. **(K)** Venn analysis of characteristics and commonality of different proteins in each group. L_C, blue light group vs. control group; M_C, Blue light with minocycline group vs. control group; M_L, blue light with minocycline group vs. blue light group.

To further investigate the death caused by blue light and the protective effect of minocycline, we used mass spectrometry. After exposure to blue light, the expression of a large amount of protein decreased due to protein hydrolysis and protein synthesis disorder. Compared with the control group, 444 proteins were downregulated and 33 proteins were upregulated in the blue light group. However, the number of downregulated proteins in the minocycline group was obviously less than that in the light group (Figures 2A–F). With the application of minocycline, the expression of proteins representing normal cell activities was upregulated, including cellular processes, environmental information processing, and genetic information processing (Figures 2G–I). Compared with the blue light group, 78 proteins were upregulated and 9 proteins were downregulated in the blue light with minocycline group (Figure 2J). Venn diagram showed differences and commonalities of proteins among groups (Figure 2K). In general, blue light exposure induces RPE cell death, and minocycline can protect RPE cells from blue light-induced death.

### Death of RPE Cells Induced by Blue Light Is Necroptosis, and Minocycline Inhibits the Process of Necroptosis

**Figure 3:**
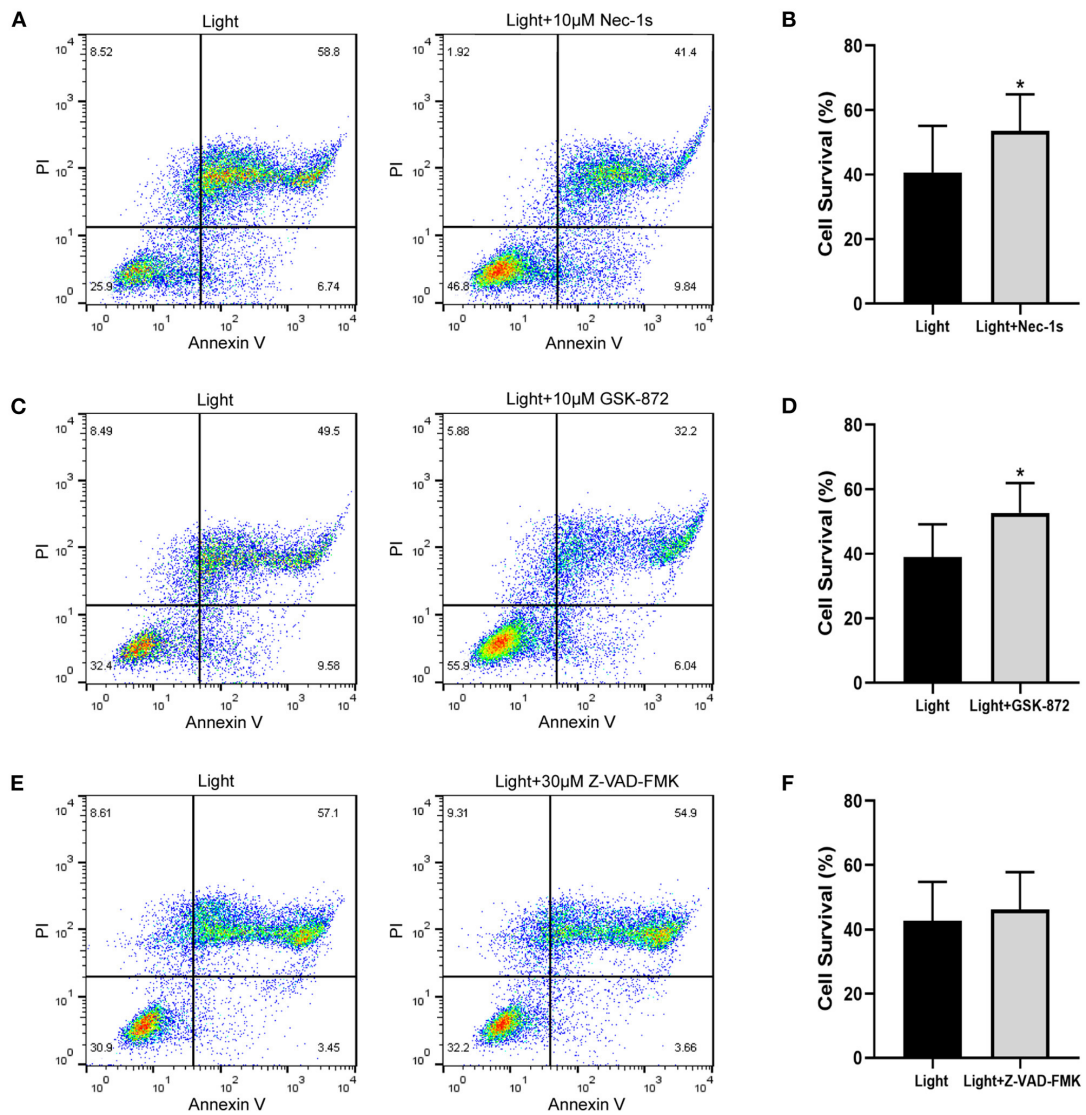
Necroptosis inhibitors improve blue-light-induced RPE cell viability, but apoptosis inhibitor does not. ARPE-19 cells received blue light exposure for 2 h and were further cultured for 2 h before follow-up experiments. **(A)** RIPK1 inhibitor Nec-1s (10 μM) improved blue-light-induced ARPE-19 cell viability. **(C)** RIPK3 inhibitor GSK-872 (10 μM) improved blue-light-induced ARPE-19 cell viability. **(E)** Pancaspase inhibitor Z-VAD-FMK (30 μM) did not improve blue-light-induced ARPE-19 cell viability. **(B,D,F)** Quantitative analysis of **(A,C,E)** (*n* = 4). *p < 0.05.

The pattern of cell death caused by blue light needs further study. Combined with the previous results of Annexin V/PI double staining, we experimentally verified the ARPE-19 cell death mode. As an inhibitor of RIPK1, Nec-1s is a more stable variant of Nec-1 and a more specific inhibitor of RIPK1 without the IDO-targeting effect (20). GSK-872 is the RIPK3 inhibitor. Both Nec-1s (*p* = 0.027) and GSK-872 (*p* = 0.025) improved cell survival (Figures 3A–D). On the other hand, the pancaspase inhibitor Z-VAD-FMK (*p* = 0.068) did not improve cell survival, which meant that the cells did not die from apoptosis but necroptosis (Figures 3E,F). Immunofluorescence experiments confirmed this point.

**Figure 4:**
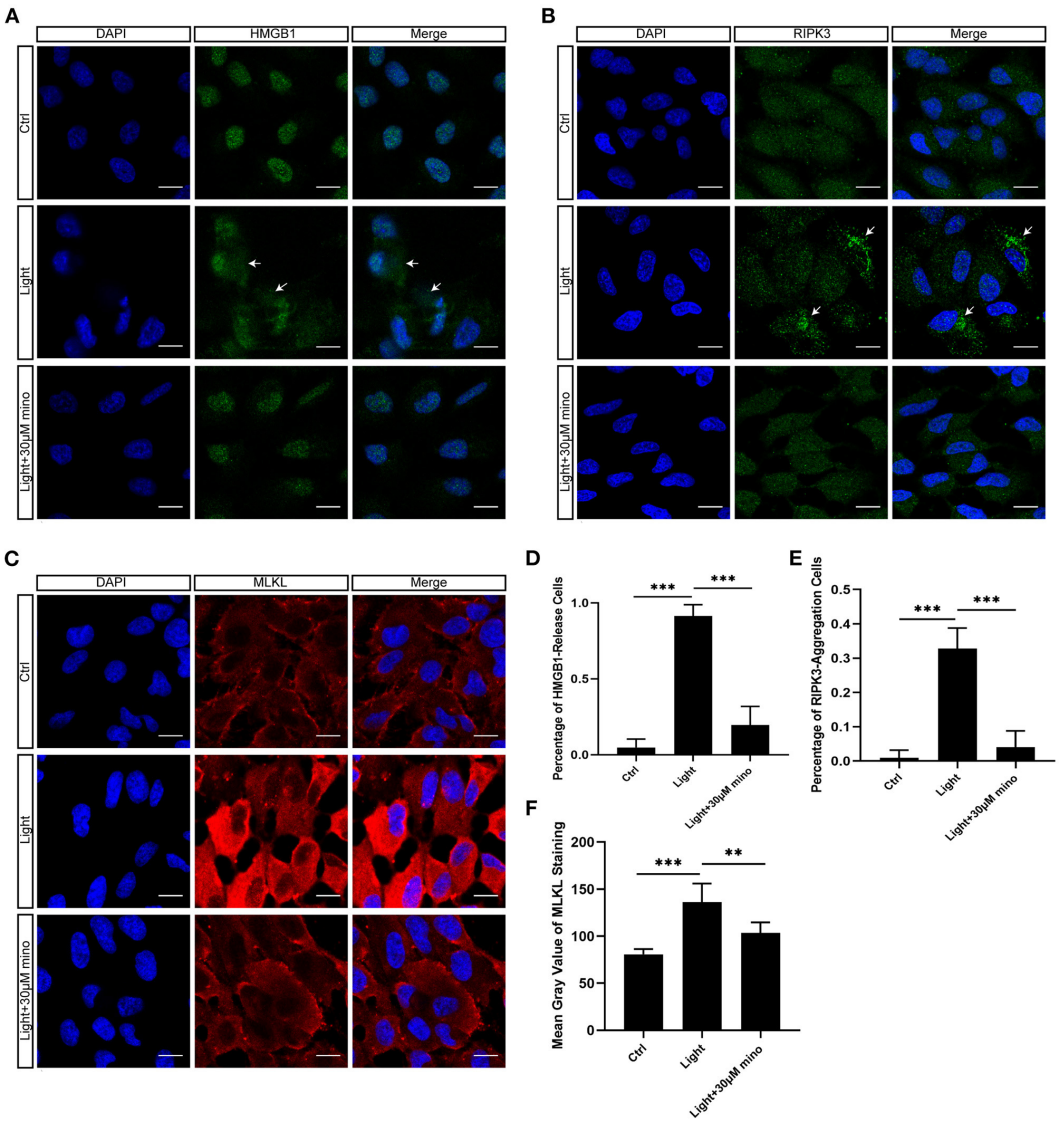
Immunofluorescence verification of necroptosis molecular hallmarks in blue-light-induced RPE *in vitro*. ARPE-19 cells were exposed to blue light for 2 h and further cultured for 2 h before immunofluorescence experiments. **(A)** Blue light caused release of HMGB1 from nuclei of ARPE-19 cells. White arrows indicated release of HMGB1 from nuclei. Minocycline treatment significantly reduced release of HMGB1 from nucleus. Scale bar: 15 μm. **(B)** Blue light caused aggregation of RIPK3 and enhancement of local fluorescence intensity in ARPE-19 cells. White arrows indicated aggregation of RIPK3. RIPK3 aggregation was evidently alleviated in light with minocycline group cells. Scale bar: 15 μm. **(C)** Compared with blue light-induced cells, MLKL staining was light in normal APRE-19 cells and slightly obvious at cell edge. After blue light exposure, fluorescence of MLKL staining was obvious and homogeneous. In blue light with minocycline group cells, fluorescence characteristics were between control group and blue light exposure group. Scale bar: 15 μm. **(D)** Quantitative fluorescence analysis of **(A)** (*n* = 6). **(E)** Quantitative fluorescence analysis of **(B)** (*n* = 6). **(F)** Quantitative fluorescence analysis of **(C)** (*n* = 6). **p < 0.01, ***p < 0.001.

As a DNA-binding nuclear protein, HMGB1 is highly conserved. HMGB1 is also a major DAMPs that is released from necrotic cells. In necrosis, HMGB1 is passively released from the nucleus into the extracellular matrix to promote inflammation (21). In apoptotic cells, HMGB1 is firmly bound to chromatin and isolated into apoptotic bodies (21, 22). We analyzed the percentage of HMGB1-release cells in different groups, and there was significant difference between groups. After exposure to blue light, HMGB1 located in the nucleus of RPE cells was released in large quantities (*p* = 0.000). This release of HMGB1 was effectively weakened with the application of minocycline (*p* = 0.000) (Figures 4A,D). In normal RPE cells, the fluorescence of RIPK3 was homogeneous. After blue light exposure, RIPK3 aggregated (*p* = 0.000), characterized by local punctiform fluorescence, suggesting the formation of necrosomes. Within the minocycline treatment, the fluorescence of RPE was similar to that of control cells (Figures 4B,E). MLKL staining was light in normal APRE-19 cells and slightly obvious at the cell edge. After blue light exposure, the fluorescence of MLKL staining was obvious and homogeneous (*p* = 0.000), indicating the recruitment and activation of MLKL. Along with the application of minocycline, the MLKL fluorescence intensity of RPE cell decreased (*p* = 0.006) (Figures 4C,F). These findings indicate that blue light can induce RPE cell necroptosis and that minocycline inhibits necroptosis and DAMPs release in RPE cells *in vitro*.

### The Mechanism of Inhibition Necroptosis by Minocycline Is Related to Mitochondria Protection

**Figure 5:**
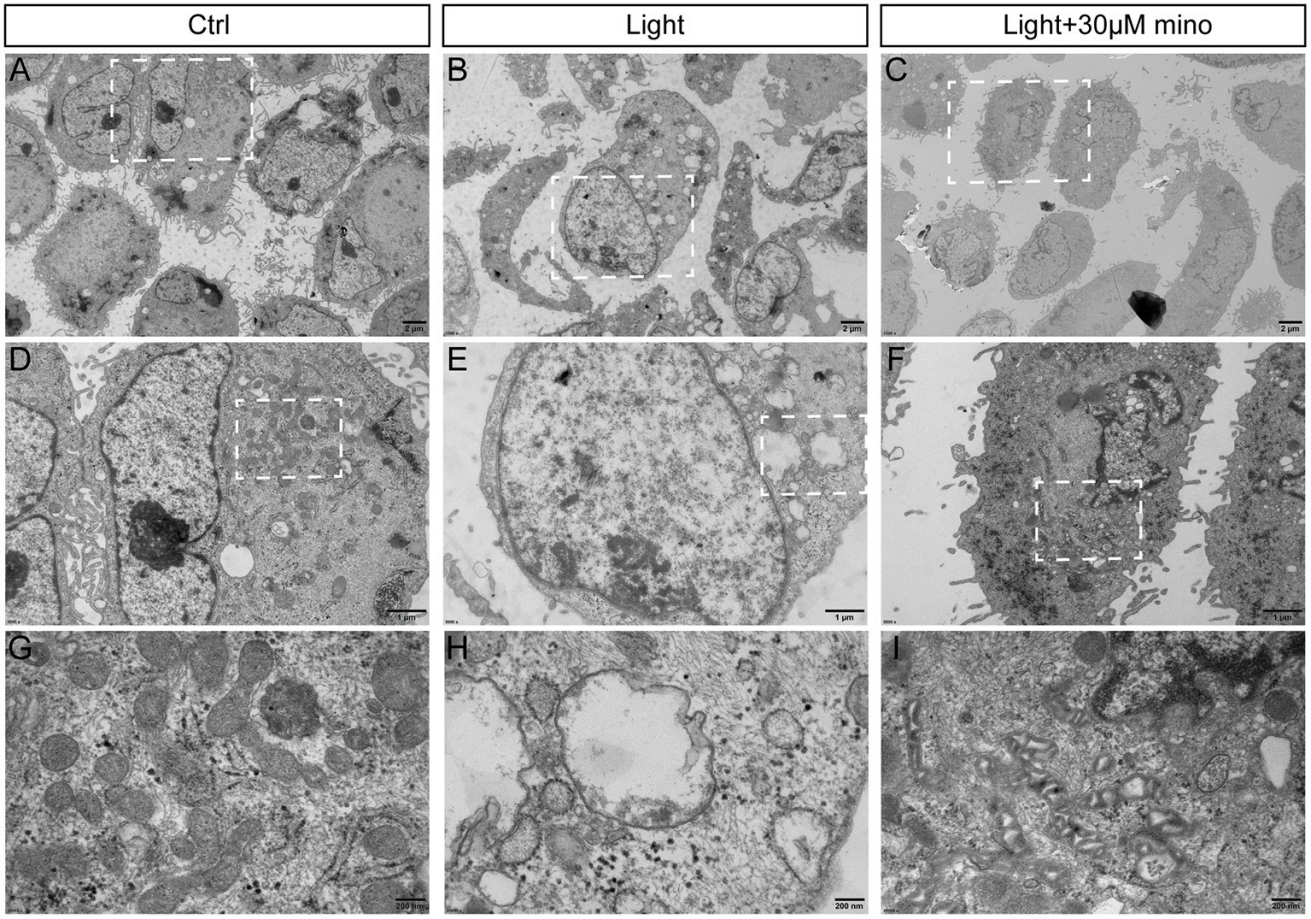
Ultrastructure of RPE cells to show blue light damage and protective effects of minocycline via transmission electron microscopy. ARPE-19 cells were exposed to blue light for 2 h and were further cultured for 2 h. **(A)** Photomicrograph of normal ARPE-19 cells. **(B)** Photomicrograph of ARPE-19 cells exposed to blue light. **(C)** Photomicrograph of ARPE-19 cells with blue light exposure and minocycline treatment. **(D–F)** Enlarged parts of white dotted boxes in **(A–C)**. In cells of blue light group, many cytoplasmic vacuoles were observed, cell membranes were damaged, and nuclear membranes were intact. **(G–I)** Enlarged views of mitochondria in white dotted boxes in **(D–F)**. Note large number of mitochondria in normal ARPE-19 cells. In blue light group, mitochondria disappeared, and vacuoles remained. Under blue light with minocycline, mitochondria existed, but mitochondrial intermembrane space was enlarged. **(A–C)** Scale bar: 2 μm. **(D–F)** Scale bar: 1 μm. **(G–I)** Scale bar: 200 nm.

Taken together, the results of flow cytometry and immunofluorescence indicated that the effect of minocycline on RPE rescue was better than that of the necroptosis inhibitors Nec-1s and GSK-872. To further explore the mechanism by which minocycline inhibits necroptosis in RPE cells, transmission electron microscopy was applied. Compared with the control group and blue light with minocycline group, the morphological changes of RPE cells of the blue light group were obvious (Figures 5A–C). RPE cells in the blue light group exhibited necrotic characteristics accompanied by microvillus disappearance, destruction of cell membranes, organelle disintegration, and vacuoles formation (Figures 5B,E). After the addition of minocycline, the illuminated cells retained some microvilli, and the cell morphology was similar to that of the control group (Figures 5D,F). Further enlarged pictures show the details of mitochondria in the three groups of cells. There were a large number of mitochondria in normal ARPE-19 cells. In the blue light group, mitochondria disappeared with vacuoles remaining. Under blue light with minocycline, the mitochondrial intermembrane space was enlarged, suggesting that mitochondrial oxidative respiration was active (Figures 5G–I). This might be why cells resisted blue light damage and avoided the formation of necroptosis.

**Figure 6:**
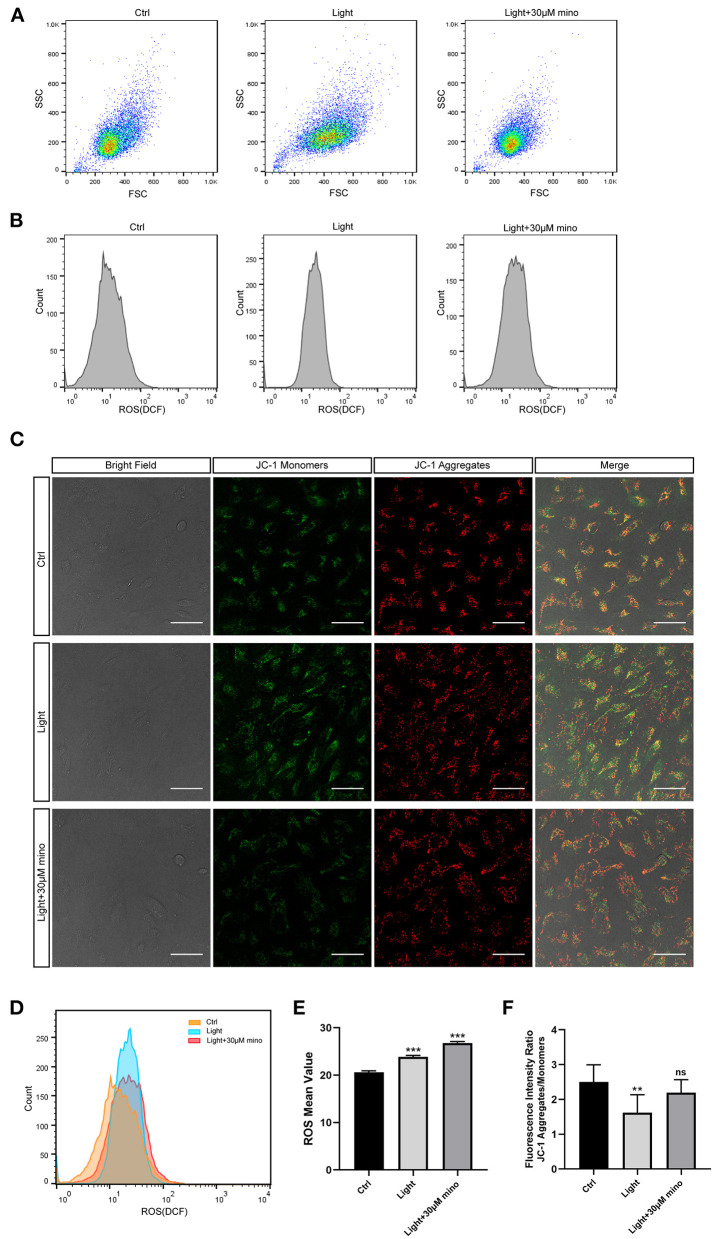
Mechanisms by which minocycline reduces light-induced RPE death. **(A)** Variations in FSCs and SSCs in ARPE-19 cells in different groups in ROS experiment. Blue light exposure time was 30 min. In blue light group, cell mass became elliptical, and FSC value moved right. In blue light with minocycline group, ARPE-19 cells were preincubated with 30 μM minocycline for 30 min before blue light exposure, and cell mass was aggregated and similar to that of control group. **(B)** ROS of ARPE-19 cells in different groups. Blue light exposure time was 30 min. **(C)** Bright field and JC-1 fluorescence images of different groups. Blue light exposure time was 1 h. Scale bar: 50 μm. **(D)** Superposition of three groups ROS diagrams in **(B)**. Curve of blue light group became sharp and moved right. Curve of blue light with minocycline group moved right. **(E)** Quantitative analysis of **(B)**. ROS in both blue light group and blue light with minocycline group was increased (*n* = 3). **(F)** Quantitative analysis of **(C)**. In blue light group, decrease in fluorescence intensity ratio suggested decrease in Δψ_m_ (*n* = 10). **p < 0.01, ***p < 0.001.

Reactive oxygen species (ROS) contain oxygen free radicals such as superoxide anion radical (${\text{O}}_{2}^{•-}$) and hydroxyl radical (^•^OH) and non-radical oxidants such as hydrogen peroxide (H_**2**_O_**2**_) and singlet oxygen (^**1**^O_**2**_) (23). ROS are mainly produced by mitochondria as byproducts of aerobic metabolism. High levels of ROS result in oxidative stress, and low levels of ROS regulate signaling pathways (24, 25). In the ROS experiment, after 30 min of blue light exposure, the RPE cell mass spread out and became elliptical in the FSC/SSC diagram, suggesting that the RPE cells were swollen and that ROS in the RPE cells increased (Figures 6A,B). The cell mass of the blue light with the minocycline group was similar to the control group, and the cell state was even better according to FSC/SSC diagram, although ROS of the RPE cells was also elevated (Figures 6A,B). In the ROS experiment, the ROS levels of the blue light group were elevated (*p* = 0.000), and minocycline did not lower the ROS levels (*p* = 0.000) (Figures 6B,D,E). Minocycline could not prevent the blue light-induced ROS increase, but the RPE cells treated with minocycline remained in good condition. This suggests that RPE cells here have a good tolerance to ROS, and ROS may not be as threatening as previously thought.

Mitochondria are the potential targets of blue light and the regulatory centers of cell death. The synthesis of ATP, uptake and storage of Ca^2+^, and generation and detoxification of ROS are inseparable from Δψ_m_ (26). The collapse of Δψ_m_ implies cell death. After 1 h of blue light exposure, RPE cells exhibited a decrease in Δψ_m_, as quantitated by the JC-1 aggregates and monomers fluorescence intensity ratio (*p* = 0.001). In the blue light with minocycline group, Δψ_m_ of RPE cells remained relatively stable (*p* = 0.133) (Figures 6C,F). The utilization of minocycline was conducive to maintaining the stabilization of Δψ_m_ and the normal physiological function of cells exposed to blue light.

In conclusion, blue light exposure can lead to an increase in ROS. However, the protective effect of minocycline does not occur by reducing ROS but stabilizing Δψ_m_. This protects the mitochondrial structure and function to maintain the normal physiological function of RPE cells and to avoid death.

### Minocycline Suppresses Necroptosis Signs in RPE Cells Caused by Blue Light *in vivo*

**Figure 7:**
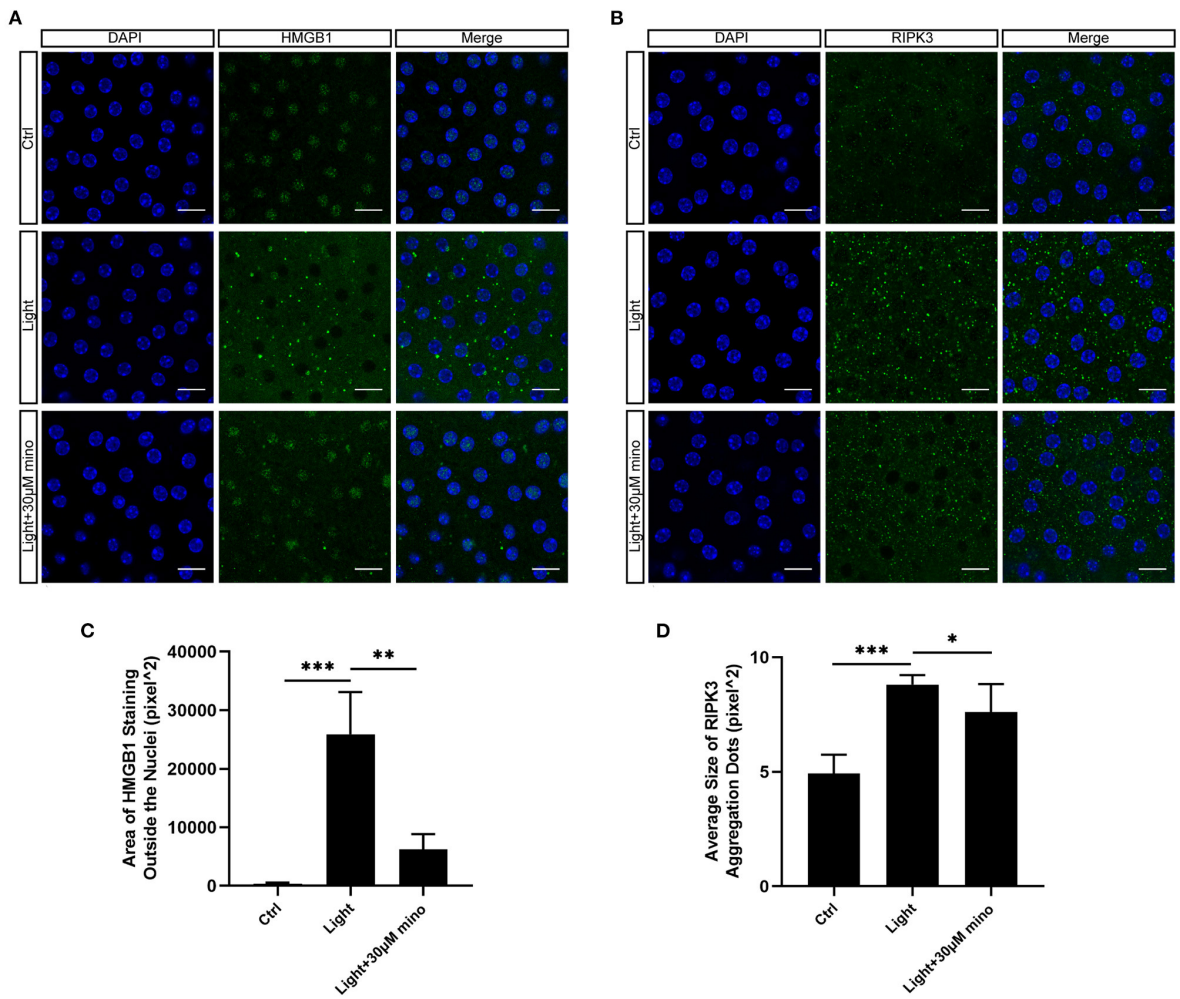
Expression of necroptosis-related proteins in RPE flatmount staining *in vivo*. **(A)** Blue light caused release of HMGB1 from nuclei of RPE cells. Minocycline treatment significantly reduced release of HMGB1 from nuclei. Scale bar: 15 μm. **(B)** Blue light caused aggregation of RIPK3 in RPE cells. Minocycline treatment alleviated aggregation. Scale bar: 15 μm. **(C)** Quantitative fluorescence analysis of **(A)** (*n* = 6). **(D)** Quantitative fluorescence analysis of **(B)** (*n* = 6). *p < 0.05, **p < 0.01, ***p < 0.001.

To analyze the influence of blue light exposure on the RPE *in vivo*, BALB/c mice were exposed to blue light. Mice that received minocycline treatment were intraperitoneally injected with minocycline. In the control group, HMGB1 was only expressed in the nucleus. In the blue light group, a large amount of HMGB1 was released from the nucleus (*p* = 0.000). In the blue light with minocycline group, minocycline reduced the release of HMGB1 from the nucleus (*p* = 0.001), although part of HMGB1 was still released into the cytoplasm (Figures 7A,C). After blue light exposure, RIPK3 staining showed punctate fluorescence enhancement, indicating the aggregation of RIPK3 (*p* = 0.000). With the application of minocycline, the aggregation of RIPK3 was slightly weakened (*p* = 0.046) (Figures 7B,D). These results indicate that DAMPs and inflammation play important roles in the process of blue light-induced RPE degeneration. Necroptosis is involved in blue light-induced RPE degeneration *in vivo*. Minocycline can alleviate necroptosis and inhibit DAMPs release.

## Discussion

In our current research, we demonstrated that blue light exposure can lead to RPE cell necroptosis and HMGB1 release and that minocycline can reduce the death of RPE cells, inhibit necroptosis, and prevent the release of HMGB1 by keeping Δψ_m_ stable.

There is significant evidence about inflammation in the process of retinal degeneration. Apoptosis is generally considered to be anti-inflammatory by withholding the signal broadcast of damaged cells. Previous studies showed that light induces the apoptosis of RPE cells (27, 28). However, different wavelengths, irradiation durations, and intensities of light can cause different levels of damage to RPE cells. At the same time, the methods to distinguish apoptosis and necrosis are limited, so the two cell death modes cannot be well-distinguished. For instance, the TUNEL assay is traditionally considered to identify apoptotic cells, but necrotic cells can also be TUNEL positive due to DNA strand breakage. Annexin V/PI staining cannot adequately distinguish between necrosis and apoptosis. Apoptosis is an ATP-dependent death mode, intracellular ATP levels keep largely unvaried until the end of the death, whereas necrosis happens under intracellular ATP depletion (29). The modes of cell death are switchable. Caspase-8 is a switch between apoptosis and necroptosis. In apoptosis, Caspase-8 suppresses necroptosis by cleaving RIPK3 (30). Study of Yang et al. showed that RPE cells presented low levels of Caspase-8 expression (31). This means that RPE cells are more prone to necroptosis when suffer damage. In addition, autophagy has been studied in blue light-induced RPE injury. Autophagy might be a protective mechanism against blue light-induced RPE damage at an early stage (32, 33) because of its homeostatic mechanism by removing faulty cellular components. Nevertheless, abnormal autophagy at the late stage of blue light-induced RPE damage might aggravate cell degeneration (32).

Necroptosis has been implicated in many human neurodegenerative diseases. Cell death and neuroinflammation motivated by necroptosis mediate the pathogenesis of these diseases (34) including ALS (35), MS (36), AD (37), and Parkinson's disease (38). In retinal neurodegenerative diseases such as RP and AMD, oxidative stress and mitochondrial dysfunction of RPE cells are observed (39–41). Hanus et al. (42, 43) found that oxidants could induce RPE cell necroptosis. This indicates that research on necroptosis is crucial for relevant pathophysiology exploration in retinal degeneration.

Necrotic cells release DAMPs, and DAMPs can cause an inflammatory response. Murakami et al. (15) showed that RIPK3 deficiency suppressed the release of DAMPs from necrotic cells and cytokine production. HMGB1 is a major DAMPs. As a prototypical alarmin, extracellular HMGB1 can activate innate immunity, and TLR4 and RAGE are the main HMGB1 receptors (44). The release of HMGB1 into the extracellular matrix can be induced by various cell stresses and diseases such as trauma, hemorrhagic shock, and sepsis (45, 46). Our study proved that blue light exposure induced HMGB1 release from RPE cell nuclei, once again proving that blue light excited RPE cell inflammation and necroptosis.

The experimental results showed that direct blue light exposure could cause necroptosis of RPE cells by damaging mitochondria, thus emphasizing the link between blue light exposure and RPE degeneration. Blue light, mitochondria, RPE necroptosis, and cell death pathways around these three factors need to be further studied.

The rescue effect of minocycline on necroptosis is much better than that of Nec-1s and GSK-872 because the rescue effect of minocycline is multifaceted. The first point is the protective function of minocycline on mitochondria. As we have proven, within minocycline, even under blue light exposure, Δψ_m_ remained relatively stable so that mitochondria could provide ATP continuously. Multiple studies have confirmed that minocycline can play an effective therapeutic role in different neurodegenerative diseases. The therapeutic properties may be due to the antiapoptotic effects of minocycline (47, 48), inhibition of key enzymes activities such as matrix metalloproteinases (MMPs) (49), inducible nitric oxide synthase (iNOS) (50), phospholipase A2 (PLA_2_) (51), inhibition of microglial activation (52), calcium chelation, and other mechanisms (17, 19). Obviously mitochondrial Ca^2+^ accumulation leads to the opening of mitochondrial permeablity transition pore. It is followed by the mitochondrial swelling and the mitochondrial membrane rupture, which results in the liberation of mitochondrial proteins, including cytochrome c (53). Overload of Ca^2+^ concentration exists at the early stage of necroptosis. Minocycline can decrease intracellular levels of Ca^2+^ and prevent the release of cytochrome c (54). Necroptosis is involved in the pathogenesis of several neurodegenerative diseases such as MS, AD, Parkinson's disease, and ALS, and it is possible that minocycline inhibits necroptosis in these neurodegenerative diseases and thus alleviates these diseases. In summary, the protective effect of minocycline may be the result of multilayered synthesis, and mitochondrial conservation is a priority in blue-light-induced RPE cell death.

The conventional view is that ROS are toxic to cells because of their high chemical reactivity. As signaling molecules and enhancing immunologic defense, ROS are also considered to be beneficial for biosystems (55, 56). This biological contradiction underlies mechanisms by which ROS are significant for the normal activities of living organisms and their senescence (55). Therefore, homeostasis of ROS is crucial to normal cell activities. The retina is a high-energy demand and highly oxygen-consuming tissue (57). The highest oxygen levels are in the choroid, whereas this descends sharply across the outer retina, forming a large gradient of oxygen (58). RPE cells perform complex and important biological functions including transporting oxygen to the outer retina, and are rich in mitochondria. Upon continuous exposure to light with intensive oxygen metabolism, RPE cells are more tolerant to oxidative stress than other cells. In our results for RPE cells in the blue light with minocycline group, although ROS of the cells were elevated, the cell state was even better, which indicated that transient ROS elevation might not lead to the death of RPE cells. In the process of blue light damage, mitochondria serve as death regulation centers, and a decrease in Δψm leads to the obstruction of ATP synthesis, which is more fatal. Long-term oxidative stress can lead to the degeneration of RPE cells; however, brief strong light exposure is more dangerous than continuous weak light stimulation.

In conclusion, our results demonstrate that blue light can induce RPE cell necroptosis and DAMPs release. Minocycline has excellent effects on inhibiting the necroptosis and DAMPs release of RPE cells under bule light illumination and can improve the survival of RPE cells remarkably by stabilizing Δψ_m_. This study provides new clues regarding the pathogenesis and treatment of retinal degeneration.

## Data Availability Statement

The original contributions presented in the study are included in the article/supplementary materials, further inquiries can be directed to the corresponding author/s.

## Ethics Statement

The animal study was reviewed and approved by Animal Care and Use Committee of Peking University First Hospital.

## Author Contributions

WS designed and implemented the experiments and drafted the manuscript. RZ participated in the model construction of blue light damage. WG and CX participated in cell experiments. LY directed the experimental design, revised the article, and provided financial support. All authors contributed to the article and approved the submitted version.

## Funding

This work was supported by the National Natural Science Foundation of China (Nos. 81470650 and 81670841).

## Conflict of Interest

The authors declare that the research was conducted in the absence of any commercial or financial relationships that could be construed as a potential conflict of interest.

## Publisher's Note

All claims expressed in this article are solely those of the authors and do not necessarily represent those of their affiliated organizations, or those of the publisher, the editors and the reviewers. Any product that may be evaluated in this article, or claim that may be made by its manufacturer, is not guaranteed or endorsed by the publisher.
